# Targeted Behavior Change Communication Using a Mobile Health Platform to Increase Uptake of Long-Lasting Insecticidal Nets Among Pregnant Women in Tanzania: Hati Salama “Secure Voucher” Study Cluster Randomized Controlled Trial

**DOI:** 10.2196/51524

**Published:** 2025-03-19

**Authors:** Trinity Vey, Eleonora Kinnicutt, Andrew G Day, Nicola West, Jessica Sleeth, Kenneth Bernard Nchimbi, Karen Yeates

**Affiliations:** 1 Department of Medicine Queen's University Kingston, ON Canada; 2 Management Sciences for Health Arlington, VA United States; 3 Kingston Health Sciences Centre Kingston, ON Canada; 4 Pamoja Tunaweza Women’s Centre Moshi United Republic of Tanzania; 5 Tanzania Education Network/Mtandao wa Elimu Tanzania Dar es Salaam United Republic of Tanzania

**Keywords:** malaria prevention, pregnant, mHealth, mobile health, short message service, behaviour change communication, long-lasting insecticidal nets, protozoan infections, parasitic diseases, vector borne diseases, randomized controlled trial, morbidity, mortality, intervention

## Abstract

**Background:**

Malaria remains a significant cause of maternal and neonate morbidity and mortality in sub-Saharan Africa. Long-lasting insecticidal nets (LLINs) represent an important component of malaria prevention and can decrease the adverse health outcomes associated with malaria infection during pregnancy. Voucher programs have been successfully implemented for a variety of initiatives across sub-Saharan Africa, including the distribution of subsidized LLINs in Tanzania. However, mobile messaging for behavior change communication (BCC), in combination with an e-voucher program, has not been explored for malaria prevention.

**Objective:**

This study aimed to assess the efficacy of mobile messaging in increasing the redemption of e-vouchers for LLINs for pregnant women and adolescents in Tanzania.

**Methods:**

This study was a blinded, 2-arm, cluster randomized controlled trial implemented in 100 antenatal health facilities in Tanzania (both urban and rural settings), with 50 clusters in both intervention and control groups. Clusters were antenatal clinics with e-voucher capabilities, with randomization stratified such that 25 urban and 25 rural clinics were randomized to each arm. Participants were pregnant females aged 13 years or older. Participants in both intervention and control groups were issued e-vouchers on their mobile phones that could be redeemed for LLINs at registered retailers within a 14-day redemption period. Participants in the intervention group received targeted BCC messages about the importance of malaria prevention and LLIN use during pregnancy, while participants in the control group did not receive BCC messages. Analyses were by intention to treat. The primary outcome was the redemption rate of e-vouchers for LLINs from retailers. Outcome measures pertain to clinic sites and individual participant-level data.

**Results:**

The study enrolled 5449 participants; the analysis included 2708 participants in the intervention arm and 2740 participants in the control arm (49 clusters in each group analyzed). There was no significant difference in the raw redemption rate of e-vouchers between pregnant participants in the intervention group (70%) and the control group (67%). Younger participants were less likely to redeem e-vouchers.

**Conclusions:**

The use of a BCC mobile messaging intervention did not result in a significant increase in LLIN uptake for pregnant individuals. However, the study shows that e-voucher distribution through nurses in antenatal clinics in partnership with local retailers is feasible on a large scale. Consideration of women and adolescents who are low-income and live in rural areas is needed for future interventions leveraging e-vouchers or mHealth technology in low-resource settings.

**Trial Registration:**

ClinicalTrials.gov NCT02561624; https://clinicaltrials.gov/ct2/show/NCT02561624

## Introduction

Malaria, a parasitic infectious disease spread by mosquitoes, was responsible for an estimated 56 million cases and 132,500 deaths in the East and Southern Africa subregion in 2020 [1]. The United Republic of Tanzania accounted for 12.8% of these total cases [1]. Pregnant women and adolescents are at heightened risk of morbidity and mortality by malaria, with 22% of pregnant women exposed to malaria in East and Southern Africa in 2020 [1]. Malaria infection during pregnancy can result in miscarriage, premature delivery, low birth weight, or death in neonates, as well as maternal anemia and maternal death [2]. Insecticide-treated nets (ITN) are recognized as a mainstay of malaria prevention, whereby trials of pregnant women in sub-Saharan Africa using ITNs during pregnancy compared with no nets reported decreased malarial infection, fewer neonate deaths before delivery, and fewer low birthweight babies [3]. Long-lasting insecticidal nets (LLINs) are a subset of ITN that retain their effective biological activity for approximately 3 years of recommended use without retreatment [4]. While malaria chemoprevention is being more widely implemented in endemic areas, especially for children and pregnant women, this is not a stand-alone strategy; LLINs remain an important component of the malaria prevention toolbox, in combination with medication [5,6].

The Tanzania National Voucher Scheme (TNVS) was a public-private partnership active between 2004 and 2014 that provided pregnant women and children with vouchers at antenatal clinics to be redeemed for ITNs and LLINs at a subsidized price [7]. The TNVS, widely known as Hati Punguzo (Swahili for “discount voucher”), contributed to an increased usage of LLINs among pregnant women, distributing 1.2 to 1.8 million LLINs per year [7]. Initially using paper vouchers, in 2011, the TNVS transitioned to electronic “e-vouchers,” allowing for enhanced tracking, real-time redemption data, and improved workflow efficiency [7]. Voucher programs have been successfully implemented in sub-Saharan Africa for a variety of initiatives, including reproductive and sexual health services, as well as agricultural interventions [8-10].

The transition of the TNVS to e-vouchers presented an opportunity for research assessing the impact of mobile health (mHealth) technology. With 495 million people subscribed to mobile phone services in sub-Saharan Africa in 2020, accounting for 46% of the region’s population, mobile phones provide a platform for widespread health interventions in low-resource settings [11].

Behavior communication change (BCC) strategies, which use targeted messaging to promote and maintain healthy behaviors, have been implemented in Africa for malaria prevention and control. In Benin, BCC messages delivered by social mobilization, radio, and flyers increased LLIN use in pregnant women [12]. Similarly, in Uganda, BCC messages spread by interpersonal communication, community mobilization, radio, and flyers increased the usage of LLIN in pregnant women [13]. However, the delivery of BCC messages through mHealth technology to increase malaria prevention efforts remains an under-researched area.

Data from 2006-2013 suggests that only 47% of women who attended an antenatal clinic accessed a net through the TNVS [7]. While the Under Five Catch Up Campaign, which distributed over 9 million free LLINs to children under 5 between 2008-2010, as well as the Universal Catch Up Campaign, which distributed over 17 million LLINs to households in 2010-2011, may have caused bed-net saturation, it remains unclear why many women were not redeeming vouchers to access LLINs before these mass campaigns [7,14]. Furthermore, LLINs typically only last up to 3 years, meaning that the protection against malaria afforded by these mass LLIN distribution campaigns is not permanent.

Despite pregnant women being at high risk of malaria morbidity and mortality, the ability of BCC messages on a mHealth platform to increase LLIN uptake for pregnant women remains uninvestigated. The Hati Salama (HASA) (Swahili for “secure voucher”) cluster randomized controlled trial aimed to test the hypothesis that BCC mobile messages targeted at pregnant women would increase redemption of e-vouchers for LLINs in regions of Tanzania with high malaria prevalence and a history of low LLIN uptake; the cluster design allowed for assessment in a real-life setting, to evaluate redemption of e-vouchers at antenatal clinic clusters. This study also aimed to determine perceived barriers to e-voucher redemption.

## Methods

### Study Design and Participants

**Figure 1:**
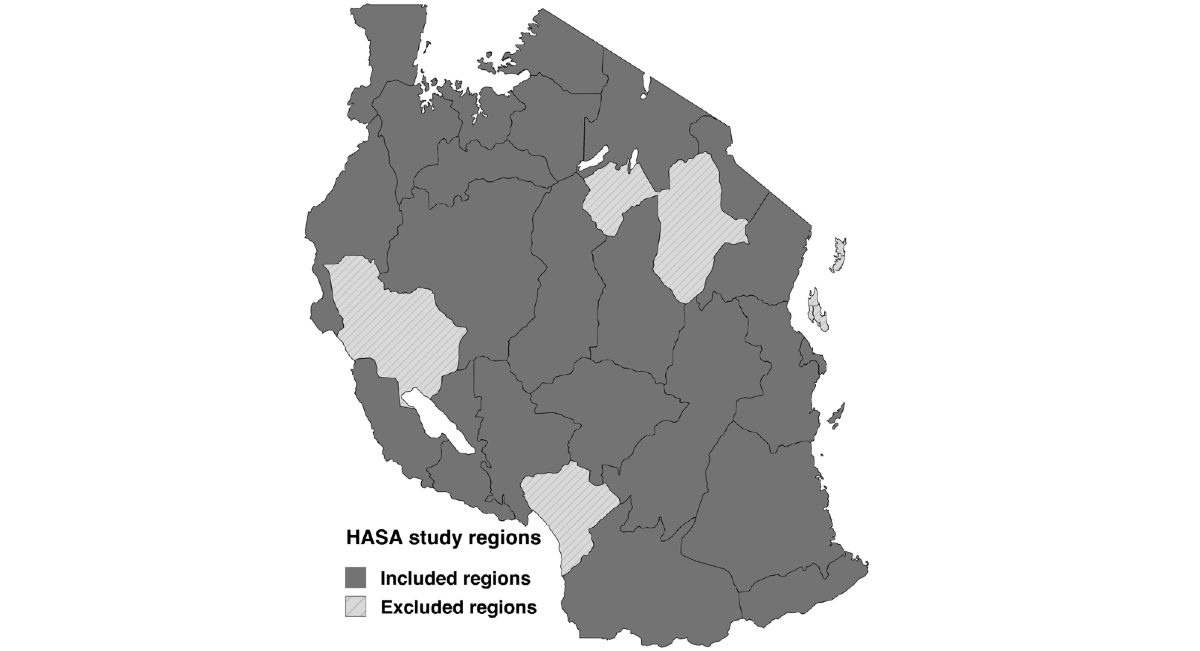
Map of the regions of Tanzania included in the Hati Salama (HASA) study.

The HASA study was a cluster randomized controlled, blinded 2-arm study implemented in 100 antenatal health facilities across Tanzania, with 50 clusters randomly allocated to the intervention group and 50 clusters to the control group, as shown in Figure 1. The study was registered on ClinicalTrials.gov (NCT02561624). The clinics selected for participation in the study were drawn from a pool of former TNVS health facilities. Study clusters were defined as clinics from both urban and rural settings with e-voucher capabilities, with an issuance of more than 175 e-vouchers per quarter but less than 70% voucher redemption of LLINs during the TNVS program.

In Figure 1, the regions highlighted in dark grey are regions of Tanzania included in the HASA study. Regions highlighted in light grey are regions excluded from the HASA study.

Participants included in the study were pregnant women and adolescents aged 13 years and older attending one of the selected antenatal health facilities. To be eligible, participants were either literate or living with someone who could assist them with reading SMS text messages. While lack of phone ownership was not a reason for exclusion, participants needed to at least have access to a proxy phone, which was recommended to be the phone of a family member, throughout the entirety of the trial.

Eligible participants who consented to participate were enrolled by trained nurses. Consent was obtained by each participant verbally and in written form by the nurses, and a signature or fingerprint of all participants was obtained. We had ethical approval to enroll participants aged 13 years and older. While most publications discuss teenage pregnancy in Tanzania for females aged 15-19, there are a number of reports that highlight teenage pregnancy for primary school-aged adolescents under and up to the age of 15 [15]. The minimum enrollment age of 13 was used as local stakeholders informed the research team of the presence of adolescents under the age of 15 attending antenatal clinics. The TNVS, the main bed net program in Tanzania, did not have a specific age limit for pregnant women, but data from that participant group informed our decision to use a low minimum enrollment age of 13 to ensure more pregnant individuals were provided the opportunity to receive an e-voucher and mHealth messaging.

Participants were ensured of the confidentiality and anonymity of the trial with regard to the information collected from them. Ethical approval was obtained from the National Institute of Medical Research Tanzania and Queen’s University Health Sciences and Affiliated Teaching Hospitals Research Ethics Board.

### Randomization and Masking

The study enrolled 50 urban and 50 rural clinics with redemption rates below 70% in the previous year. Each site was asked to enroll at least 50 participants. Randomization was stratified such that 25 urban and 25 rural clinics were randomized to each arm. The statistician prepared the randomization list before the start of the trial using clinic codes of the 50 urban and 50 rural participating sites without knowing which clinic the codes referred to. Randomization was performed by randomly sorting 25 intervention and 25 control assignments within each of the 50 rural and 50 urban clinics by a computer-generated sequence. All health care workers and relevant stakeholders, including nurses, were blinded to their group assignment, as were participants and investigators, although participants would presumably become aware of their assignment after receiving their first BCC SMS message. Intervention group nurses and control group nurses were trained separately, and the control and intervention clinics were located large distances from each other to minimize contamination.

### Procedures and Interventions

Participants in both the intervention and control groups were issued LLIN e-vouchers on their mobile phones by nurses upon enrollment into the study. E-vouchers could be redeemed for a high-quality LLIN at registered retailers for a discounted price; while the original cost of LLINs was approximately US $8, participants with an e-voucher were only required to pay a co-payment of 500 Tanzanian shillings (at the time of the study, equivalent to approximately US $0.25). All women attending antenatal clinics enrolled in the study were offered an e-voucher through the HASA program, regardless of whether they provided informed consent to participate in the trial and receive BCC messages. Women who accepted e-vouchers but did not participate in the study were not included in the analyses, but all women enrolled in the study were included.

The study undertook a phased approach with rolling enrolment during the period from July to December 2015, whereby each site had 12 weeks to complete recruitment, and each individual participant had 14 days for e-voucher redemption, starting from when they were enrolled. Participants attending one of the 50 clinics allocated to the intervention group received targeted BCC messages on their registered mobile phones at programmed intervals during the 14-day period in which the e-voucher was active. Messages contained health promotion and sensitization information on the importance of malaria prevention and LLIN use during pregnancy, in addition to reminders to redeem the e-voucher for an LLIN at a registered retailer (Textbox 1). Participants attending one of the 50 clinics randomized into the control group did not receive any targeted BCC messages on their phones during the 14 days following e-voucher issuance. As the allocation of clinics to intervention and control groups was random and blinded, all participants were told by study nurses that they may or may not be receiving SMS messages during the 14 days following issuance. Each participant also had a case report form completed by a nurse, collecting information including the participant’s age, marital status, number of pregnancies, number of children, previous LLIN ownership, and education level.

Following voucher expiration (14-day period), all participants who did not redeem their e-vouchers in both the intervention and control groups received a follow-up phone survey asking them to indicate the reason why they did not redeem the voucher for an LLIN. All participants were analyzed through intention to treat in the arm their cluster was randomized to, regardless of whether they redeemed their e-voucher or not.

**Content of SMS behavior communication change (BCC) messages sent to participants (translated from Swahili to English).**

- Insecticide-treated bed nets are proven to prevent malaria when used correctly when sleeping in your home.
- Insecticide-treated bed nets should always be used when you or your children sleep.
- Malaria can make young children very sick and can cause death.
- Malaria is a very serious illness among pregnant women. It can have dangerous consequences for the woman and her unborn child, including death.
- Your small children should always sleep under a bed net, and bed nets should be replaced every 5-7 years or as soon as they are in poor condition.
- You have recently received a voucher that can be redeemed for a bed net, but you have not yet redeemed this voucher.
- The net voucher you received at the clinic can be redeemed at any participating shop (Duka or Duka la Dawa) that provides bed nets for Hati Punguzo.
- This is a reminder that you received a bed net voucher 1 week ago, and you have not yet redeemed it to receive your bed net.
- This is a reminder that you received a bed net voucher 10 days ago, and you have not yet redeemed it to receive your bed net.

### Outcomes

The prespecified primary outcome was the redemption rate of e-vouchers for LLINs from retailers in regions with high malaria prevalence and a history of low uptake. Outcomes were measured using participant-level redemption data available through the electronic platform. The data displayed how many participants (with appropriate code and phone numbers) redeemed their e-vouchers for an LLIN within the 14-day period from each clinic and the exact times the transactions took place; outcome measures pertain to cluster sites and individual participants. A subsequent manuscript will examine perceived causes and barriers to nonredemption by a structured phone survey of participants who did not redeem their e-vouchers within the allotted 14-day period [16]. In addition, with regard to longer-term outcomes, the trial serves as a pilot study to test the overall effectiveness, security, and sustainability of a mobile platform design and its potential for delivering other private-sector health solutions in the region.

### Statistical Analysis

When calculating the sample size, a baseline voucher redemption rate of 50% was estimated, with a 10% increase to 60% considered minimally clinically important. Furthermore, it was assumed that the intracluster correlation would be 0.1 and that each site would enroll 50 participants. Under these assumptions, 47 sites per arm would provide over 80% power at a 2-sided α=.05. To allow for potential loss of sites or underrecruitment at some sites, 50 sites per arm were enrolled. This calculation did not account for our analysis adjusting for reach (urban vs rural) and baseline site redemption rates, which could increase power slightly.

The redemption rates were compared between arms using a generalized estimating equation with a compound symmetric (ie, exchangeable) working correlation structure. A binomial distribution and an identity link were used so that adjusted risk differences could be estimated. However, it was confirmed that all *P* values remained nearly identical if the logit link was used. Adjusted risk differences were based on the estimate of an intervention indicator variable. The model also included the site level baseline (baseline referring to the previous year) redemption rate as a linear covariate and the site reach (urban vs rural) indicator variable. *P* values and confidence intervals were based on the Wald method using empirical standard errors. Since the primary analysis only adjusted for site-level factors, nearly identical results were obtained in a sensitivity analysis performed at the site level using a regression model with arm, baseline redemption rate, and reach as independent variables and the observed site redemption rates as the dependent variable. Sensitivity analyses were also performed, excluding one outlier site and including only participants who were known to have had their own mobile phones.

The ANOVA estimator was used to estimate the intracluster correlation coefficient of e-voucher redemption separately by the arm with 95% CIs estimated by the nonparametric percentile bootstrap based on 1000 bootstraps for each arm. Our bootstrap selected entire clinics rather than individual participants [17].

The research team attempted to identify predictors of e-voucher redemption based on our preselected list of site and participant characteristics. First, the redemption rate was compared by characteristic. Subsequently, the aforementioned generalized estimating equation approach was used to identify significant predictors of e-voucher redemption. A multivariable model was selected, including predictors of e-voucher redemption based on a backward selection with an exit criterion of *P*<.15.

All analysis was performed using SAS (version 9.4; SAS Institute).

### Role of the Funding Source

This Project is supported by Grand Challenges Canada. Grand Challenges Canada is funded by the Government of Canada and is dedicated to supporting Bold Ideas with Big Impact.

### Ethical Considerations

This study was conducted in accordance with ethical standards for human subject research (Declaration of Helsinki). Before initiation, the study protocol and study forms were reviewed and given approval by the National Institute for Medical Research in Tanzania approval (certificate number NIMR/HQ/R.8a/VOL./IX/1750) and Queen’s University Health Sciences and Affiliated Teaching Hospitals Research Ethics Board institutional review board in Canada (approval number 6013000).

Participants were informed that their participation and responses would be completely confidential and no identifying information about them would be recorded and stored. Each participant was given a copy of the informed consent document. Consent was allowed in both verbal and written formats after the participant was given enough time to review the document and ask questions or discuss any concerns in a private setting with a research team member. Participants were informed throughout the consent process and the duration of the study that participation was voluntary, they may withdraw from the study at any time without consequence, and that they would still receive an e-voucher for redeeming a long-lasting insecticidal net (LLIN) even if they decided not to participate and withdrew. No compensation was given for participation in this study.

## Results

**Figure 2:**
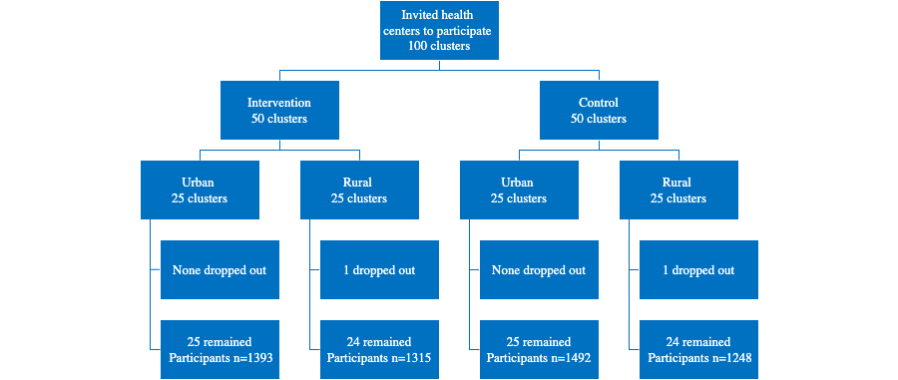
CONSORT diagram of clinic and participant flow. CONSORT: Consolidated Standards of Reporting Trials.

The trial took place between July and December 2015. A total of 25 rural and 25 urban clinics were randomized to each study arm. During the study timeline, each clinic had a 12-week period to complete the recruitment of at least 50 participants. Each participant had 14 days for e-voucher redemption starting from when they were enrolled. One rural clinic in the control arm and 1 rural clinic in the intervention arm dropped out of the study for administrative reasons after randomization but before enrolling any participants. This left a total of 49 clinics in each arm, as shown in Figure 2. In total, 5449 participants from the randomized sites consented to participate in the trial. One of the consented participants was excluded from the analysis because their e-voucher redemption status was uncertain, leaving 2708 participants in the intervention arm and 2740 participants in the control arm. Site and participant characteristics were well-balanced between arms (Table 1). In the year before the study, the average (SD) redemption rate at the participating sites was 54% (SD 12%) in control groups and 54% (SD 13%) in intervention groups. (range 3%-68%). In the control groups, the average age of participants was 26.4 (6.2) years old (range 13-49), the median parity was 2, 87% (2372/2740) were married, and 62% (1693/2740) were known to have their own phones (no proxy phone). In the intervention groups, the average of participants was 26.4 (6.2), the median parity was 2 (IQR 1-4), 85% (2311/2708) were married, and 62% (1687/2708) were known to have their own phones. Where data for marital status, education, or proxy phone status is missing, this indicates that this data was not collected by antenatal nurses, or participants declined to provide this information (Table 1).

**Table 1:** Baseline site and participant characteristics.

				Control		Intervention
**Clinic characteristics**				Clinics (n=49)		Clinics (n=49)
	***Location***					
		Rural, n (%)	24 (49)		24 (49)	
		Urban, n (%)	25 (51)		25 (51)	
	Baseline redemption rate (%), mean (SD)		54 (12)		54 (13)	
**Participant characteristics**				Participants (n=2740)		Participants (n=2708)
	Age, mean (SD)		26.4 (6.2)		26.4 (6·2)	
	Parity, median (IQR)		2 (1-4)		2 (1-4)	
	Number of children, median (IQR)		1 (0-3)		1 (0-3)	
	Number of LLINs^a^ owned, median (IQR)		1 (1-2)		1 (1-2)	
	Number of LLINs accessed through TNVS^b^, median (IQR)		0 (0-1)		0 (0-1)	
	***Marital Status, n (%)***					
		Missing	87 (3)		99 (4)	
		Single	281 (10)		298 (11)	
		Married	2372 (87)		2311 (85)	
	***Education, n (%)***					
		Missing	128 (5)		166 (6)	
		None	335 (12)		327 (12)	
		Primary	1736 (63)		1637 (60)	
		Secondary	447 (16)		468 (17)	
		College	94 (3)		110 (4)	
	***Proxy, n (%)***					
		Missing	663 (24)		691 (26)	
		No	1693 (62)		1687 (62)	
		Yes	384 (14)		330 (12)	

^a^LLINs: long-lasting insecticidal nets.

^b^TNVS: Tanzania National Voucher Scheme.

**Figure 3:**
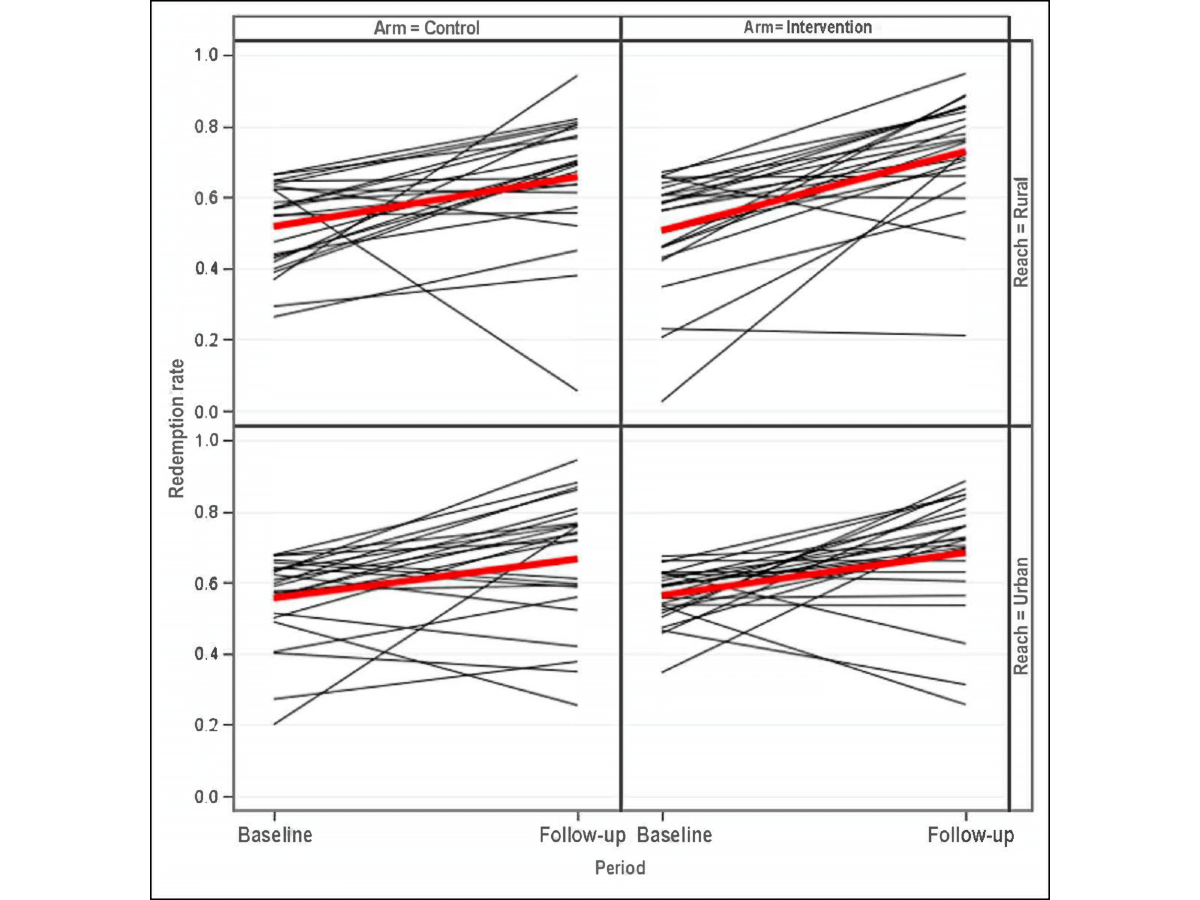
Site-specific redemption rates. Black lines depict site specific rates and bold red line shows overall mean rate.

Figure 3 displays the within-site-specific change in redemption rates from the previous year compared with the rate during the study. Generally, the rates increased. The actual site-specific counts are presented in Table 2. The one notable outlier was the 24th rural site in the control group (Table 2). This site had a baseline redemption rate of 62% yet had only a 6% (1/18) redemption rate during the study. This site had substantially fewer participants than the next lowest accruing site, which had 33 participants (all other sites accrued at least 50 participants as planned) and had a much lower redemption rate than the next lowest site, which had a rate of 21%. After querying this site, it was found that they had administrative problems, which hindered accrual and redemption. Our primary analysis includes all sites as randomized, but a sensitivity analysis excluding this one outlying site was performed.

In Figure 3, the black lines depict site-specific rates, and the bold red line shows the overall mean rate.

Overall, the raw redemption rate in the intervention arm was 70% compared with 67% in the control arm. The difference after controlling for the sites’ reach (urban vs rural) and the sites’ baseline redemption rates was 4% (95% CI –2% to 10%; *P*=.19). After excluding the one outlying site, the raw rates were 70% and 68% in the intervention and control arms, respectively, and the adjusted difference was 3% (95% CI –3% to 9%; *P*=.31). The intracluster correlation of e-voucher redemption status (without adjusting for covariates) was 0·12 (95% CI 0.05 to 0.15) in the intervention arm and 0·13 (95% CI 0.7 to 0.16) in the control arm. In a prespecified subgroup analysis, the 3380 (62%) participants who were known to have their own phones were included. In this subgroup, the raw redemption rates in the intervention and control arm were 71% and 69%, respectively, with an adjusted risk difference of 3% (95% CI –3% to 9%; *P*=.27).

Table 3 compares the site and participant characteristics between participants who did and did not redeem their e-vouchers. Of note, people who redeemed their vouchers were 0.7 years older than those who did not. E-voucher redemption status was lower among participants where marital status, education, or proxy status was missing; however, there was no difference in e-voucher redemption rates among known education levels, marital status, or proxy status. Where proxy status was missing, this indicates that this data was not collected by antenatal nurses or participants declined to provide this information (Table 3). On average, participants who redeemed their e-vouchers came from sites with a baseline rate of 2% (*P*=.04) higher than participants who did not redeem their e-vouchers. In fact, aggregated to the site level, the correlation between the previous redemption rate compared with the study redemption rate was *r*=0.23 (*P*=.03), and after excluding the one outlier site (with only 18 participants and 1 redemption), the correlation became *r*=0.27 (*P*=.007). Parity, number of children, number of LLINs owned, number of LLINs accessed through TNVS, and clinic reach were not significantly different between participants who did and did not redeem their e-vouchers.

All participant characteristics were missing for 186 participants, an additional 55 were missing parity, an additional 108 were missing education, and an additional 950 were missing proxy. For categorical variables, the total number of missing values is displayed, but for continuous variables, the missing values have been excluded.

Table 4 provides the single predictor and multiple predictor regression results. The multiple predictor regression included all predictors that were significant at *P*<.15 by backward selection. From the multiple predictor model, it was determined that for every 10% increase in the sites’ baseline redemption rate, the study redemption rate increased by 3% (95% CI 0%-6%, *P*=.03), and for every decade of age, the redemption rate increased by 4% (95% CI 2%-5%, *P*<.001).

**Table 2:** Number of vouchers issued and redeemed for each site. Rows are sorted by the descending number of vouchers issued.

	Intervention arm						Control arm					
Site	Urban		Rural			Urban					Rural	
	Previous, %	Follow-up, n/N (%)	Previous, %	Follow-up, n/N (%)	Previous, %			Follow-up, n/N (%)		Previous, %		Follow-up, n/N (%)
1	53	18/70 (26)	46	49/65 (75)	61			53/72 (74)		44		36/63 (57)
2	61	47/65 (72)	42	55/62 (89)	56			54/71 (76)		42		50/62 (81)
3	59	44/63 (70)	46	53/60 (88)	62			36/69 (52)		62		38/62 (61)
4	50	54/61 (89)	35	33/59 (56)	40			38/68 (56)		66		46/60 (77)
5	52	41/59 (69)	61	34/57 (60)	56			54/68 (79)		47		41/59 (69)
6	54	44/58 (76)	66	54/57 (95)	67			48/67 (72)		39		38/55 (69)
7	61	45/57 (79)	58	48/56 (86)	50			49/66 (74)		55		35/55 (64)
8	54	30/56 (54)	66	27/56 (48)	40			22/63 (35)		43		38/54 (70)
9	63	24/56 (43)	3	40/55 (73)	64			48/63 (76)		55		30/54 (56)
10	66	37/56 (66)	56	39/55 (71)	64			37/63 (59)		57		43/54 (80)
11	46	46/55 (84)	43	38/54 (70)	57			41/62 (66)		57		38/53 (72)
12	56	31/55 (56)	50	37/54 (69)	68			46/60 (77)		57		41/53 (77)
13	59	40/55 (73)	56	41/54 (76)	20			44/58 (76)		65		43/53 (81)
14	59	41/54 (76)	61	46/54 (85)	55			41/57 (72)		37		49/52 (94)
15	59	37/54 (69)	63	46/54 (85)	63			49/57 (86)		43		35/52 (67)
16	63	34/54 (63)	64	42/54 (78)	66			34/57 (60)		63		27/52 (52)
17	67	38/54 (70)	21	34/53 (64)	58			32/54 (59)		65		34/52 (65)
18	63	32/53 (60)	59	38/53 (72)	63			51/54 (94)		26		23/51 (45)
19	56	42/52 (81)	23	11/52 (21)	66			33/54 (61)		64		41/51 (80)
20	63	44/52 (85)	61	39/51 (76)	27			20/53 (38)		29		19/50 (38)
21	66	44/52 (85)	46	40/50 (80)	60			46/53 (87)		40		35/50 (70)
22	47	16/51 (31)	58	41/50 (82)	59			42/52 (81)		66		41/50 (82)
23	51	44/51 (86)	66	33/50 (66)	49			13/51 (25)		58		21/33 (64)
24	35	38/50 (76)	67	42/50 (84)	51			21/50 (42)		62		1/18 (6)
25	47	36/50 (72)	—^a^	—	68			44/50 (88)		—		—
Arm total by strata	56	947/1393 (68)	51	960/1315 (73)	56			996/1492 (67)		52		843/1248 (68)
Arm total	54	1907/2708 (70)				54			1839/2740 (67)			

^a^Not available.

**Table 3:** Voucher redemption status by baseline site and participant characteristics.

Characteristics				Redeemed, clinics (n=49); participants (n=3746)			Not redeemed, clinics (n=49); participants (n=1702)		*P* value^a^
**Clinic characteristics**									
	***Location, n (%)***								
		Rural	1803 (70)			760 (30)			.52
		Urban	1943 (67)			942 (33)			
	Baseline redemption^b^ (%), mean (SD)				54 (12)	52 (13)		.04	
**Participant characteristics**									
	Age^b^, mean (SD)				26.6 (6.2)	25.9 (6)		<.001	
	Parity^b^, median (IQR)				2 (1-4)	2 (1-4)		.06	
	Number of children^b^, median (IQR)				2 (0-3)	2 (0-3)		.07	
	Number of LLINs^c^ owned^b^, median (IQR)				1 (1-2)	1 (1-2)		.42	
	Number of LLINs accessed through TNVS^b,d^, median (IQR)				0 (0-1)	0 (0-1)		.96	
	***Marital status, n (%)***								<.001
		Missing	64 (34)			122 (66)			(.58)
		Single	406 (70)			173 (30)			—^e^
		Married	3276 (70)			1407 (30)			—
	***Education, n (%)***								.005
		Missing	140 (48)			154 (52)			(.80)
		None	468 (71)			194 (29)			—
		Primary	2373 (70)			1000 (30)			—
		Secondary	627 (69)			288 (31)			—
		College	138 (68)			66 (32)			—
	***Proxy, n (%)***								
		Missing	879 (65)			475 (35)			.008
		No	2373 (70)			1007 (30)			(.34)
		Yes	494 (69)			220 (31)			—

^a^*P* values estimated from generalized estimating equations with redeemed (yes or no) as the dependent variable. For categorical variables, missing was included as a category in the top *P* value, while the second *P* value (in parentheses) excludes missing values.

^b^Continuous predictors are modeled as linear, but the number of LLINs owned and accessed through TNVS was truncated at 5 since less than 1% of participants had a value >5.

^c^LLINs: long-lasting insecticidal nets.

^d^TNVS: Tanzania National Voucher Scheme.

^e^Not applicable.

**Table 4:** Regression models for predictors of voucher redemption rates.

				Single predictor models					Selected model^a^		
Characteristics				Risk difference (95% CI)		*P* value		Risk difference (95% CI)			*P* value
**Clinic characteristics (%), median (IQR)**											
	***Location***										
		Rural	2 (–4 to 9)		.51		5 (–1 to 11)			.12	
		Urban	Referent								
	Baseline redemption (per 10%)		3 (0 to 6)		.04		3 (0 to 6)			.03	
**Participant characteristics (%), median (IQR)**											
	Age (per decade)^b^		4 (2 to 6)		<.001		4 (2 to 5)			<.001	
	Parity (per 1)^b^		1 (0 to 1)		.06		—^c^			—	
	Number of children (per child)^b^		1 (0 to 1)		.07		1 (0 to 1)			.11	
	Number of LLINs^d^ owned (per 1)^b^		0 (–1 to 1)		.43		—			—	
	Number of LLINS accessed through TNVS^e^ per 1)^b^		0 (–1 to 1)		.96		—			—	
	Single vs married		–1 (–5 to 3)		.58		—			—	
	***Education (%), median (IQR)***				.8		—			—	
		None	1 (–3 to 4)		—		—			—	
		Primary	Referent		—		—			—	
		Secondary	–1 (–4 to 2)		—		—			—	
		College	–2 (–8 to 4)		—		—			—	
	***Proxy (%), median (IQR)***										
		No	Referent		.34						
		Yes	–3 (–5 to 2)		—		—			—	

^a^The selected model excludes 186 participants with missing values of the selected variables.

^b^Continuous predictors are modeled as linear, but the number of LLINs owned and accessed through TNVS was truncated at 5 since less than 1% of participants had a value >5.

^c^Not applicable.

^d^LLINs: long-lasting insecticidal nets.

^e^TNVS: Tanzania National Voucher Scheme.

## Discussion

### Principal Findings

This cluster randomized trial aimed to assess the effectiveness of mHealth technology in the uptake of LLINs for pregnant women in Tanzania. It was anticipated that targeted BCC mobile messages would increase the redemption of e-vouchers for LLINs in regions of Tanzania with high malaria prevalence and a history of low LLIN uptake. However, our results demonstrate no significant difference in the redemption of e-vouchers between pregnant women in the intervention group that received BCC mobile messages and those in the control group that did not receive mobile messages.

### Comparison With Previous Work

The existing literature using mHealth messaging as a tool for malaria prevention and control in sub-Saharan Africa shows a combination of both successful and unsuccessful interventions. There are several studies investigating the feasibility of mHealth technology as a malaria surveillance tool in sub-Saharan Africa that have shown encouraging results [18-20]. Evidence also suggests that mobile messaging improved antimalarial drug supply management in Tanzania, improved Kenyan health care workers’ adherence to malaria treatment guidelines, increased adherence to malaria rapid diagnostic test results in Nigeria, and increased adherence to artemisinin-based combination therapy regiments in Ghana [21-24]. There are also several mHealth studies with conflicting results. A cluster randomized trial sending mobile messages about artemether-lumefantrine treatment to retail staff in Tanzania drug shops found some improvement in dispenser knowledge but no difference in participant adherence, suggesting that increased knowledge of retail staff did not translate to increased adherence in participants [25]. Furthermore, a randomized controlled trial in Ghana sending mobile messages to caregivers of children with malaria under 5 years of age found increased posttreatment return to the health facility but no impact on treatment adherence [26]. It was suggested that this could be due to adherence levels already being high in both groups, meaning that mobile messages were unnecessary. Similarly, it is possible that pregnant women in Tanzania have significant baseline knowledge that LLIN use in pregnancy is important, regardless of educational mobile messaging.

In the HASA study, site and participant characteristics were well-balanced between intervention and control groups, and characteristics including parity, number of children, education levels, and marital status did not impact redemption. Notably, younger participants were less likely to redeem their e-vouchers, and for every decade of age, the redemption rate increased by 4%. Previous literature from Tanzania suggests that individuals in the age group 30-49 had the greatest knowledge of malaria, potentially explained by having more life experience than younger individuals [27]. Together, this may suggest a need for interventions directly targeting pregnant women below the age of 30 years.

Despite a null result, both groups experienced an increase in LLIN uptake from baseline during the trial. As such, one possible explanation for the null result is that nurses at participating antenatal clinics contributed to the increased uptake of LLINs in both groups. Despite nurses being blinded to their intervention group, antenatal nurses likely played a role in encouraging LLIN uptake. Antenatal clinic nurses were involved in orientation and extensive training for the HASA trial (nurse training described in subsequent manuscript), which may have prompted them to provide additional information about malaria and motivate pregnant women in both groups to follow through on e-voucher redemption upon enrollment into the study [16]. This would be consistent with previous findings in Tanzania that for pregnant women, knowledge of malaria correlates with ITN uptake [28]. While this represents an overall positive health outcome resulting from the trial implementation, it is a type of contamination bias from a clinical trial perspective, whereby the effects of the intervention were received by members of the control group and possibly shifted the outcome of the control group in the same direction as the intervention group [29]. Contamination bias is a potential problem for clinical trials of educational and behavioral interventions due to the ease of transferring intervention knowledge; in this case, information about the importance of LLINs and malaria prevention could be inadvertently shared with participants in the control group by nurses, thereby changing the behavior of the control group participants and impacting the results of the trial [29].

The current prevailing practices for increasing ITN uptake in pregnant women focus on a multipronged approach. This includes integrating ITN provision and counseling into antenatal care visits, engaging and educating the community about the importance of ITNs, strengthening health care systems by ensuring the availability and distribution of ITNs to health care facilities, and training health care workers on ITN promotion and counseling techniques [30,31]. Due to the importance of increasing widespread LLIN use in pregnancy to prevent malaria and its morbidity and mortality, and given the significant body of evidence for the use of mobile messaging to promote healthy behavior change in low- and middle-income country contexts, the authors feel that there is still significant equipoise in this area of research.

### Strengths and Limitations

A major strength of the HASA study was the distribution of e-vouchers from antenatal clinics. Antenatal care coverage in Tanzania has been above 90% of pregnant women for over 2 decades, with 98% receiving care from a skilled provider in 2015-2016 [32]. Over half of pregnant women in Tanzania had 4 or more antenatal facility visits during the year of the trial, providing ample opportunity for nurses to provide them with LLIN e-vouchers at visits [32].

An additional strength of the study was that it was a large, robust study. A total of 5448 women were enrolled in the study, and 3746 e-vouchers were redeemed. One can assume that 5.5 lives per year are saved for every 1000 children with an LLIN in the household [33]. With 3746 e-vouchers redeemed by pregnant women with an average of 2 children each already and a new baby (assuming all pregnancies resulted in a live birth), the HASA study protected 11,238 children and saved approximately 62 lives, which is a success in terms of trial implementation.

A major challenge encountered in this study was the low penetration of mobile phone ownership in women at numerous study facility sites, with only 62% (1687/2708) of participants possessing their own phones. This represents a barrier for researchers and health program implementers considering using a mHealth strategy to improve access to health care and health education for rural-dwelling low-income women. Participants whose phone proxy status was missing or not available were less likely to redeem their e-vouchers. This low penetration reflects the gap in gender equity in mobile phone ownership. In sub-Saharan Africa, women are 13% less likely than men to own a mobile phone; literacy, digital skills, and affordability are the largest barriers to phone access [34]. Women in rural areas, particularly Maasai women, may have been disadvantaged in terms of the trial as they are less likely to own a phone; cell coverage in these rural areas can also be particularly poor.

Unexpected outages of cellular network services in many parts of Tanzania during the study represented another challenge. Clinic nurses reported network outages and the resulting inability to activate e-vouchers while a participating woman was at the clinic. The investigators are aware that some women who sought to participate were unable to either receive or redeem their e-vouchers due to connectivity issues. Given that cellular coverage is increasing in rural areas and network reliability is consistently improving, perhaps in a few years, these issues will be less prevalent for mHealth studies.

Previous findings suggest that in Tanzania, women of low socioeconomic status have a lower uptake of ITN vouchers than women of higher socioeconomic status since there is still some expense required to obtain a net [35]. The voucher redemption rate for ITNs in Tanzania is influenced by socioeconomic status and co-payment cost [36]. This disparity for women of lower socioeconomic status may be amplified using e-vouchers, as these women may be less likely to have their own mobile phones or live in an area with reliable connectivity. Despite the co-payment cost being relatively low, this finding suggests the need for interventions targeted at low-income women.

In Tanzania, the literature has indicated past challenges with the misuse of ITN vouchers. A study investigating a paper discount voucher scheme in Tanzania in 1999 suggested that some vouchers were not being used for the intended population (pregnant women and young children), likely resulting in increased usage of nets in men and older children [35]. As the HASA study measured e-voucher redemption, a limitation is an inability to determine whether the LLINs were used by the intended population following e-voucher redemption. Furthermore, in 2013, there was evidence of fraudulent e-voucher activities by some clinic and retail staff, whereby staff could create vouchers for fake beneficiaries so that LLINs could be subsequently sold on the commercial market [7]. For the HASA study, the potential of e-voucher fraud was addressed by a redesign of the e-voucher to create a more secure system. Components of the redesign included a unique identifier for all pregnant women, random barcode usage activated once at retailers, periodic reports to the clinic in charge to verify the numbers issued, the ability of participants to provide SMS feedback to confirm whether a voucher code was received, and a log indicating SMS delivery confirmation or errors causing delivery failure.

### Conclusions

The HASA study has demonstrated that the use of a mHealth intervention for BCC mobile messaging does not result in a significant increase in LLIN uptake for pregnant women. However, the use of an e-voucher distributed by nurses in antenatal clinics in partnership with local retailers is feasible on a large scale and holds promise for other health interventions. Future studies evaluating e-vouchers or mHealth initiatives should take into consideration the barriers to phone ownership experienced by those who are most vulnerable, especially among women who are low-income and live in rural areas.

## Appendix

Multimedia Appendix 1 CONSORT eHealth Checklist.

## Abbreviations

BCC: behavior change communication
HASA: Hati Salama
ITN: insecticide-treated nets
LLIN: long-lasting insecticidal net
mHealth: mobile health
TNVS: Tanzania National Voucher Scheme

## Data Availability

The datasets generated and/or analyzed during this study are available from the corresponding author on reasonable request.
